# Differences in the adsorption of enzymes onto lignins from diverse types of lignocellulosic biomass and the underlying mechanism

**DOI:** 10.1186/1754-6834-7-38

**Published:** 2014-03-14

**Authors:** Fenfen Guo, Wenjing Shi, Wan Sun, Xuezhi Li, Feifei Wang, Jian Zhao, Yinbo Qu

**Affiliations:** 1 State Key Laboratory of Microbial Technology, Shandong University, Ji-nan City, Shandong Province 250100, China

**Keywords:** Enzyme adsorption, Diverse types of biomass, Lignin property, S/G ratio

## Abstract

**Background:**

Non-productive cellulase adsorption onto lignin has always been deemed to negatively affect the enzymatic hydrolysis of lignocellulosic feedstocks. Therefore, understanding enzyme-lignin interactions is essential for the development of enzyme mixtures, the processes of lignocellulose hydrolysis, and the genetic modification of lignocellulosic biomass and enzymes. In this work, we examined the properties of six lignins from diverse types of lignocellulosic biomass (aspen, pine, corn stover, kenaf, and two *Arabidopsis* lines, wild-type and SALK mutant of *fah1*) to determine the mechanism of differences in their adsorption of enzymes.

**Results:**

We found that lignin sources affected enzyme adsorption using structural features, such as functional groups and lignin composition. Guaiacyl (G) lignin had a higher adsorption capacity on enzymes than syringyl (S) lignin. The low S/G ratio and high uniform lignin fragment size had good correlations with high adsorption capacity. A higher content of phenolic hydroxyl groups and a lower content of carboxylic acid groups resulted in stronger adsorption affinity for corn stover lignin (CL) than for kenaf lignin (KL) and aspen lignin (AL). The lower amount of aliphatic hydroxyls that reduced hydrophobic interactions could explain the higher adsorption capacity of pine lignin (PL) than CL. Enzyme activity assays, as well as the hydrolysis of Avicel, phosphoric acid-swollen cellulose (PASC), and holocellulose, were performed to study the behaviors of mono-component enzymes that resulted in adsorption. We found that cellobiohydrolase (CBH) and xylanase were adsorbed the most by all lignins, endoglucanase (EG) showed less inhibition, and β-glucosidase (BG) was the least affected by lignins, indicating the important role of carbohydrate-binding module (CBM) in protein adsorption.

**Conclusion:**

Lignin sources affect enzyme adsorption using structural features and lignin composition, such as S/G ratio, carboxylic acid, aliphatic hydroxyl, and phenolic hydroxyl. For mono-component enzymes, the adsorption capacity decreased in the order CBH, xylanase > EG > BG. These investigations revealed the difference in lignin properties between diverse biomass and adsorption capacity of enzymes to lignins, and the possible underlying mechanism. The results can also serve as a reference for the genetic modification of lignocellulosic biomass and enzymes.

## Background

The conversion of lignocellulosic materials into bioethanol has drawn worldwide attention because of concerns about the depletion of fossil fuels. Two major constituents of lignocellulosic biomass, cellulose and hemicellulose, can be converted to fermentable sugars. However, another main component, lignin, has been always deemed to have a negative impact on the saccharification of lignocellulosic feedstocks by physically barring and unproductively adsorbing hydrolytic enzymes [1-3].

Lignin is an aromatic cell wall polymer in vascular plants. It encrusts and glues the network of cell wall carbohydrates together, stiffening the cell wall structure. Its biosynthesis occurs through the radical coupling of the lignin precursors, coniferyl, sinapyl, and *p*-coumaryl alcohol, giving rise to a random sequence of guaiacyl (G), syringyl (S), and hydroxyphenyl (H) subunits in the polymer, respectively. The sequence varies from plant to plant, and the ratio of G, S, and H units significantly differ in softwood, hardwood, and grass species. However, three main types are recognized: softwood lignin is mainly composed of G units (G-type), whereas both G and S units are abundant in hardwood (GS-type). In addition to G and S units, H units are also present in grasses and compression wood lignin (GSH-type). However, to date, no study has been conducted about the adsorption of enzymes onto lignins from these diverse types of biomass. Whether the difference in lignin composition affects adsorption capacity is also unclear. Moreover, the genetically engineered biomass with modified S/G ratio in lignin, which serves as a more promising sugar source for ethanol production, is being actively studied [4,5]. Thus, further understanding between lignin composition and enzyme adsorption could guide the genetic modification of biomass.

The adsorption of enzymes onto isolated lignins has been widely studied, and most of the studies involve different enzymes or lignins extracted from pretreated materials [1,2,6,7]. In a previous study, cellulases and xylanases have been found to be significantly affected by lignin, with β-glucosidase (BG) being the least affected [1]. Carbohydrate-binding module (CBM) has been identified to play an important role in protein adsorption [2]. The adsorption capacity has been found to differ among various lignins from pretreated materials [6]. Using the good correlation between the hydrolysis yields of the pretreated lignocellulose and Avicel containing the isolated lignin, Nakagame *et al*. [8] reported that native differences in lignin may be the reason for the differences in their inhibitory properties. However, the mechanism on how it affects the inhibitory hydrolysis was not explained. Pan [9] also found that the hydrolysis inhibition was dependent on lignin sources and the structural features of lignin, like how functional groups were important for enzyme-binding or the enzyme-interfering capacity of lignin. However, comparison studies on the adsorption capacities of native lignins from diverse types of biomass that consider their physicochemical properties have not been conducted. Therefore, it is of interest to study them in terms of total protein adsorption and mono-component enzyme adsorption to help elucidate the fundamentals behind protein adsorption. Proper understanding of the lignin structures that promote enzyme adsorption could help provide a reference for the genetic modification of lignocellulosic biomass.

It is well known that hydrophobic interactions have been identified to be a major driving force for protein adsorption onto lignin [1,6]. For functional groups, which affect lignin-enzyme interactions, a low carboxylic acid group content and a higher phenolic hydroxyl content in lignins could result in the increased protein adsorption capacity [2], and the data by Berlin *et al*. [1] also indicate that the polydispersity index (PDI) is inversely related to the interaction of the polymer with the protein.

The present study focuses on the adsorption of the enzyme from *Penicillium oxalicum* JU-A10-T onto lignin preparations from diverse types of biomass (softwood, hardwood, and herbaceous plants) and two *Arabidopsis* lines (wild-type and SALK mutant of *fah1*). The differences in the properties of lignins, such as chemical functionalities, molecular weight, PDI, and composition, were also determined to investigate their potential connections to enzyme adsorption affinity.

## Results and discussion

### Adsorption of cellulase onto lignins from different types of lignocellulosics

**Figure 1:**
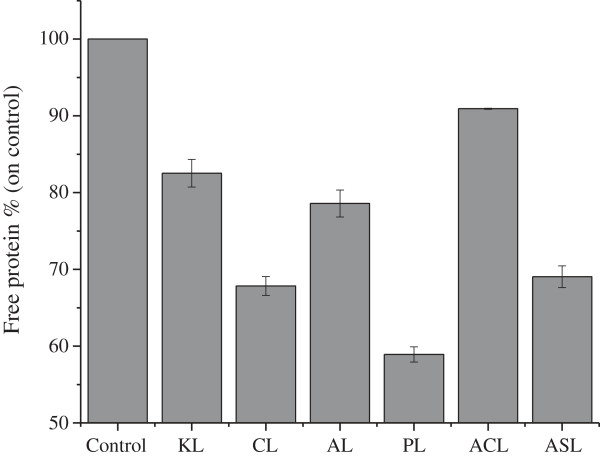
**The changes in the protein content in the supernatant after the adsorption by lignins.** Samples that lack lignin served as control. ACL, *Arabidopsis* Col-0 lignin; AL, aspen lignin; ASL, *Arabidopsis* SALK mutant 063792 lignin; CL, corn stover lignin; KL, kenaf stalk lignin; PL, pine lignin.

To compare the adsorption of extracellular proteins in crude enzymes from the liquor fermentation of *P. oxalicum* to that in lignins from diverse types of biomass, 5.4 mg protein/g lignin mixed systems were made, because evident change and difference in the protein band could be clearly observed in the reaction system, according to a previous study (data not shown). The changes in protein content resulting from the adsorption by lignins are shown in Figure 1, and the visible differences in protein band change could also be observed in SDS-PAGE (data not shown). Obviously, there were significant differences in the adsorption capability of cellulase onto lignins among the various lignocellulosic materials. Corn stover lignin (CL) and pine lignin (PL) had the highest adsorption affinity on the enzyme, and aspen lignin (AL) had stronger adsorption capacity than kenaf lignin (KL). The adsorption capacity decreased in the order PL > CL > AL > KL. For the two *Arabidopsis* lines, *Arabidopsis* SALK mutant 063792 lignin (ASL) adsorbed significantly more protein than *Arabidopsis* Col-0 lignin (ACL).

### Inhibitory effects of lignins on mono-component enzymes

**Figure 2:**
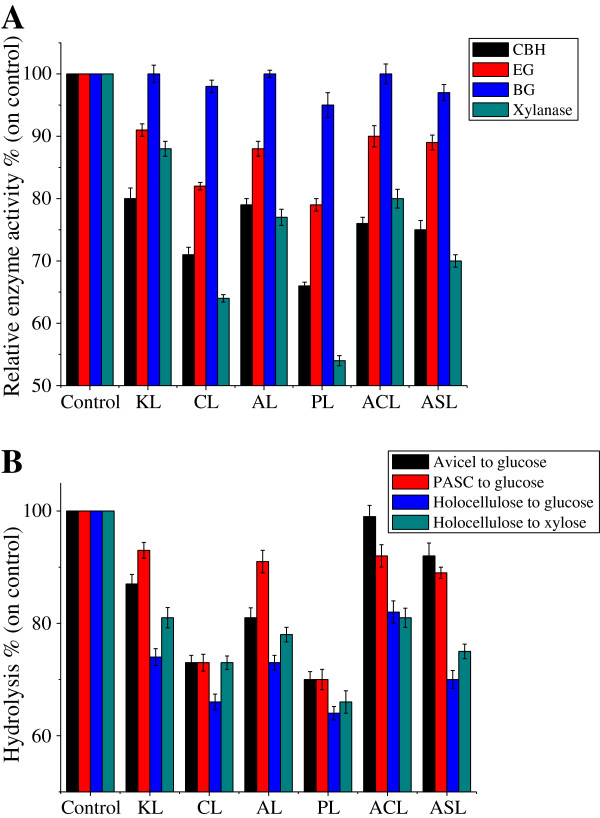
**(A) The changes of enzyme activities and (B) the inhibition effect of lignins on the enzymatic hydrolysis of various celluloses.** Samples without lignin served as control. ACL, *Arabidopsi*s Col-0 lignin; AL, aspen lignin; ASL, *Arabidopsis* SALK mutant 063792 lignin; BG, β-glucosidase; CBH, cellobiohydrolase; CL, corn stover lignin; EG, endoglucanase; KL, kenaf lignin; PASC, phosphoric acid-swollen cellulose; PL, pine lignin.

Protein quantification provides an overview of the enzyme adsorption capacities of the lignin preparations. To gain insight about the adsorption of prominent enzymes in the protein preparations studied, activity assays for cellobiohydrolase (CBH), endoglucanase (EG), BG (cellulase), and xylanase (hemicellulase) were performed (Figure 2A). Similarly, the behavior of the mono-component enzyme that resulted in adsorption could also be observed in the hydrolysis of Avicel and phosphoric acid-swollen cellulose (PASC), representing the change of CBH and EG, respectively, and holocellulose, representing the change of xylanase and total cellulase (Figure 2B). These show that CL and PL had the most significant decrease in enzyme activities, and CBH and xylanase were adsorbed the most by all lignins. EG was less inhibited, which may be attributed to the difference in protein structure, like hydrophobic sites. BG, which had no CBM, was the enzyme least affected by the lignins (Figure 2). According to the results of the hydrolysis of holocellulose, total cellulase was found to have a stronger inhibition effect on lignins than xylanase. Berlin *et al*. [1] also reported that cellulases and xylanases are significantly affected by lignin, and BG was the enzyme that was least affected. In cellulase, CBH is found to have a higher affinity to lignins than EG. Lower lignin-binding was detected when using the enzyme without CBM and the linker domain, thus, CBM was found to play an important role in protein adsorption [2], which could explain the least inhibition of BG by lignin.

### Lignin properties and possible mechanism for adsorption differences

To understand the reasons for the differences in their adsorption capacities of protein, the properties of the lignins were investigated using Fourier transform infrared spectroscopy (FTIR), nuclear magnetic resonance (NMR) spectra, and gel permeation chromatography (GPC) methods.

#### *FTIR analysis of lignins*

Signal assignment and relative intensities in the FTIR spectra of lignins are shown in Table 1. Compared with KL and AL, CL had much lower intensities at 2,938, 2,879, 2,845 cm^-1^ (C-H vibrations in the methyl and methylene groups), and 1,462 cm^-1^ (C-H deformations in the methyl groups and aromatic ring vibrations), suggesting a higher H unit content and lower S unit content, because H units had no OCH_3_ groups, and S units had two OCH_3_ groups linked to aromatic rings, confirming that CL could be referred to as a GSH lignin. Moreover, in CL, the significantly higher intensity at 830 cm^-1^ and the additional band at 1,168 cm^-1^ (typical for GSH lignin, except when indicating an antisymmetric C-O stretching of ester groups) also confirms this point [10]. The low intensity at 1,123 cm^-1^ (aromatic C-H deformation in S ring) and high intensity at 1,032 cm^-1^ (aromatic C-H in plane deformation G + S) suggests a lower S/G ratio in CL than KL and AL. KL had higher intensities at 2,938, 2,879, 2,845, 1,462, and 1,123 cm^-1^, and lower adsorption at 1,268 cm^-1^ than AL, suggesting higher S unit content, lower G unit content, and higher S/G ratio. AL (hardwood) is known to be GS-type, which the intensity at 1,168 (none) and 833 cm^-1^ (0.15) also confirms. The zero adsorption at 1,168 cm^-1^ and the little intensity at 833 cm^-1^ (0.20) means that KL had very low H unit content, which indicates that KL is between hardwoods (GS) and grasses (GSH) in terms of lignin. The lower intensity at 2,879, 1,462, 1,123, and 830 cm^-1^ and the almost zero adsorption at 1,329 cm^-1^ reveals the G-type of PL.

**Table 1:** Signal assignment and relative intensities in FTIR spectra of lignins

**Assignment**	**Peak cm**^**-1**^	**KL**	**CL**	**AL**	**PL**	**ACL**	**ASL**
O-H stretching in aromatic and aliphatic hydroxyl groups	3,446	2.00	1.69	1.62	1.54	5.56	5.25
C-H vibrations in the methyl and methylene groups	2,938	0.56	0.42	0.49	0.55	0.70	0.64
	2,879	0.36	0.30	0.33	0.26	0.34	0.30
	2,845	0.31	0.22	0.29	0.30	0.40	0.36
Nonconjugated carbonyl groups (C = O stretch)	1,724	0.58	1.12	0.69	0.46	-	-
Aromatic skeleton vibration	1,594	1.17	1.25	1.05	0.73	-	-
	1,507	1.00	1.00	1.00	1.00	1.00	1.00
	1,422	0.79	0.66	0.73	0.40	0.55	0.43
C-H deformations and aromatic ring vibrations (methyl)	1,462	1.01	0.89	0.95	0.61	0.54	0.47
Phenolic hydroxyl groups	1,384	0.47	0.61	0.46	0.14	-	-
Syringyl ring breathing, C-O stretch	1,329	0.45	0.54	0.51	0.01	0.09	0.02
Guaiacyl C-O units	1,268	0.52	-	0.82	0.64	0.74	0.89
Aromatic methyl ether bridges	1,225	0.86	1.13	0.83	0.38	1.00	0.90
	1,083	-	1.08	-	0.25	1.08	1.05
C-O stretch in ester group	1,168	-	1.01	-	-	0.80	-
Aromatic C-H deformation in syringyl ring	1,123	1.85	1.38	1.37	0.47	1.54	1.26
Aromatic C-H in plane deformation G + S	1,032	1.10	1.46	0.98	0.64	1.21	1.33
Aromatic C-H out of plane C-H out of plane in position 2 and 6 of S, and in all positions of H	830	0.20	0.37	0.15	0.07	0.21	0.15

The relative intensity was calculated as the ratio of the intensity of the band to the intensity of band at 1,507 cm^-1^. ACL, *Arabidopsis* Col-0 lignin; AL, aspen lignin; ASL, *Arabidopsis* SALK mutant 063792 lignin; CL, corn stover lignin; FTIR, Fourier transform infrared spectroscopy; G, guaiacyl; H, hydroxyphenyl; KL, kenaf lignin; PL, pine lignin; S, syringyl.

Compared with KL and AL, CL had much higher intensities at 1,225, 1,083, and 1,168 cm^-1^ (ester group), indicating the lower amount of carboxylic acid groups, which has negative correlation with protein adsorption affinity by reducing the hydrophobic interactions [6,11]. In previous studies, the increase in the amount of phenolic hydroxyls in lignin has been linked to increased enzyme binding/inhibition capacity [12]. A study using lignin model compounds confirms that phenolic hydroxyl groups, which are important and necessary sites for the protein-adsorption and protein-precipitation capacity of tannins and lignins, play a key role in lignin-enzyme interactions [9]. Ximenes *et al*. [13] have also reported an increase in the inhibition and/or deactivation effect caused by the addition of soluble phenolic compounds, which could inactivate enzymes by reversibly or irreversibly complexing them. Rahikainen *et al*. [2] found that the higher content of phenolic hydroxyls in steam explosion pretreated lignins compared to the untreated lignins is a possible explanation of the higher affinity of the studied cellulases on steam explosion pretreated lignins. In this study, CL was found to have a significantly higher phenolic hydroxyl (1,384 cm^-1^) content compared to KL and AL (Table 1), which is a possible factor that contributes to higher adsorption capacity. However, these did not fit well for PL, which had the lowest intensities at 1,225, 1,083, 1,168, and 1,384 cm^-1^, showing that there were other factors that caused the highest adsorption affinity on the enzyme.

Compared to ACL (wild-type), ASL had much lower intensities at 1,329 and 1,125 cm^-1^, and higher intensity at 1,268 cm^-1^, indicating a lower S/G ratio. ASL was derived from *Arabidopsis* mutant 063792, in which the encoding gene of ferulate-5-hydroxylase (F5H), *fah1*, was SALK mutated to avoid S-type lignin formation. F5H is a potential regulatory step in the determination of lignin monomer composition, which catalyzes an irreversible hydroxylation step in the pathway that diverts ferulic acid away from G lignin biosynthesis and toward sinapic acid and S lignin [14]. Thus, ASL had no S lignin and reduced S/G ratio because the total lignin content was the same in the two *Arabidopsis* lines. The S/G ratio was 0.12 and 0.02 (almost zero) for ACL and ASL, respectively, estimated using Abs_1329_/Abs_1268_[15]. The low S/G ratio, which is one of the few differences between ACL and ASL, could be recognized as the main factor of the significant difference in the enzyme adsorption. And this result also shows that G lignin has higher adsorption affinity than S units. This may also be a reason why PL and CL showed higher affinity onto the protein compared to KL and AL.

#### *NMR analysis*

**Figure 3:**
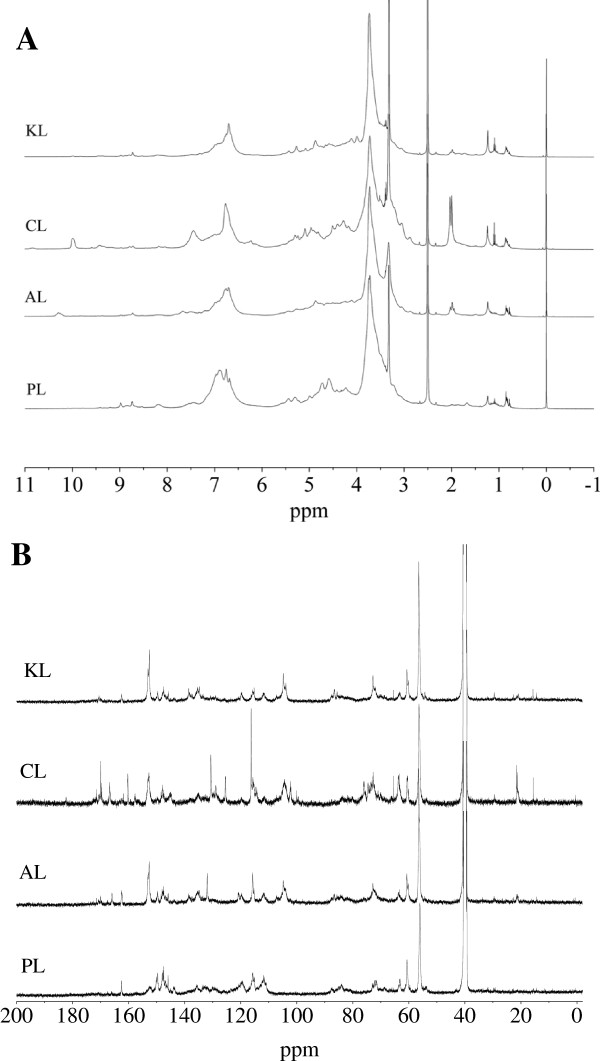
**(A) The **^**1**^**H and (B) **^**13**^**C-NMR spectra of lignins.** AL, aspen lignin; CL, corn stover lignin; KL, kenaf lignin; NMR, nuclear magnetic resonance; PL, pine lignin.

To further investigate the structural features of the lignins, the ^1^H and ^13^C-NMR spectra of the milled wood lignins (MWL) from the different lignocellulosic materials were recorded (Figure 3). Assignment of signals in the ^1^H and ^13^C-NMR spectra of lignin are shown in Table 2 and Table 3, respectively. In ^1^H-NMR spectra (Figure 3A), the CL had significantly strong adsorption at 7.4 (G lignin), which is in accordance with the FTIR analysis that CL had a high content of G lignin. PL had no adsorption and CL had higher adsorption at aliphatic -OH (2.0 and 1.9 ppm). A lower content of aliphatic hydroxyls in lignin reportedly affects higher enzyme binding/inhibition capacity by increasing the surface hydrophobicity [1], which probably explains the higher adsorption capacity of PL than that of CL.

**Table 2:** **Assignment of signals in **^**1**^**H-NMR spectra of lignin**

**ppm**	**Assignments**
10.4	H in -OH
7.4	H in G
6.9	Hα (α,β in conjugated double bonds)
4.8	Hγ (β-5, β-1, β-β, β-O-4)
4.0	H_β_ (β-O-4)
3.8	HOMe (aromatic)
3.6	
3.3	H_2_O
2.6	DMSO
2.0	H in aliphatic -OH
1.9	
1.2	H in aliphatic -OH of high-shielding effect
1.1	
0.8	
0.0	Unknown

DMSO, dimethyl sulfoxide; G, guaiacyl; H, hydroxyphenyl; NMR, nuclear magnetic resonance.

**Table 3:** **Assignment of signals in **^**13**^**C-NMR spectra of lignin**

**ppm**	**Assignments**
172.6	C = O
170.9	
166.3	-COO- in FA and CA esters
161.6	C-4 in FA and CA esters
159.9	C-4 in H
152.1	C-3/C-5 in S etherified, C-3/C′-3 in 5-5′ etherified
147.2	C-3/C-5 in S, C-4/C-3 in G
145.2	C-α in FA and CA esters, C-4 in G not etherified
130.0	C-2/C-6 in FA and CA ethers
124.7	C-α in coniferyl alcohol
115.8	C-3/C-5 in FA and CA esters, C-3/C-5 in H, C-5 in G
113.7	C-2 in G
103.6	C-2/C-6 in S
83.0	C-_β_ in β-O-4, C-α in β-5 and β-β
72.3	C-α in β-O-4
66.2	C-γ in β-5
62.8	C-5 in xyl internal unit
60.1	C-γ in β-O-4
55.7	-OCH_3_
28.9	-CH2-(C5-CH2-C5)
21.0	-CH3 in acetyl group
15.2	γ-methyl in *n*-propyl side chain

CA, *p*-coumaric acid; FA, ferulic acid; G, guaiacyl; H, hydroxyphenyl; NMR, nuclear magnetic resonance; S, syringyl.

The ^13^C-NMR spectra included the region of aromatic carbons between 103 and 162 ppm (155 to 142 aromatic C-O, 142 to 124 aromatic C-C, and 124 to 102 aromatic C-H) and the aliphatic carbon region (90 to 60 ppm) [16]. In the ^13^C-NMR spectra result (Figure 3B), the significantly strong adsorptions at 172.6, 170.9 (C = O), 166.3 (-COO-), 159.9, 128 (H units), 147.2 (G + S), and 145.2 ppm (G) in CL were in accordance with the FTIR analysis. Esterified *p*-coumaric acid, as a precursor of H units and a usual content in monocotyledonous plants, was clearly indicated by strong signals at 167, 161, 130.5, 125.5, and 116 ppm, further supporting the GSH-type for CL [16]. The significant adsorption at 21.0 ppm (CH_3_- in acetyl groups) in CL also supports the significant adsorption at 15.2 ppm (γ-methyl in *n*-propyl side chain), like the analysis of FTIR and ^13^C-NMR (more C = O). No adsorption at 152.1 and 103.6 ppm (S units) and the higher intensity at 147.2 and 145.2 (G units), further supports the G-type for PL.

**Figure 4:**
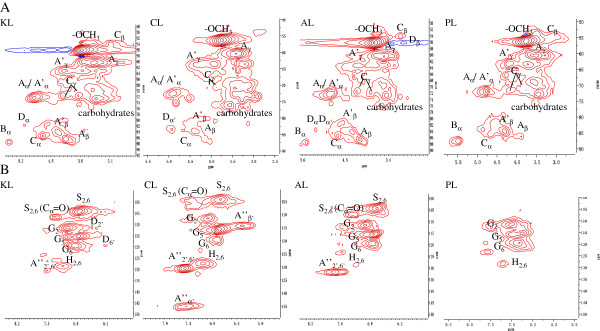
**(A) Expanded side chain (**δ_**C**_**/**δ_**H **_**50 to 90/2.5 to 5.5) and (B) aromatic (**δ_**C**_**/**δ_**H **_**100 to 150/5.5 to 8.5) regions in the HSQC spectra of lignins.** AL, aspen lignin; CL, corn stover lignin; HSQC, heteronuclear single quantum coherence; KL, kenaf lignin; PL, pine lignin.

Two-dimensional heteronuclear single quantum coherence (2D-HSQC) NMR has been capable of providing important structural information on lignin, for example, substructures (inter-coupling bonds). The HSQC spectra showed three regions corresponding to aliphatic (δ_C_/δ_H_ 10 to 50/0.5 to 2.5), lignin side chain (δ_C_/δ_H_ 50 to 90/2.5 to 5.5), and aromatic (δ_C_/δ_H_ 100 to 150/5.5 to 8.5) regions [10]. The assignments of ^13^C-^1^H cross-signals in the HSQC spectra are shown in Table 4. The aliphatic (nonoxygenated) region showed no signals with structural information (except for the presence of acetate signals at δ_C_/δ_H_ 20.7/1.74), and therefore is not discussed in detail. In the side-chain region, methoxyls (OCH_3_, δ_C_/δ_H_ 56.2/3.73) and side-chains in β-O-4 substructures were the most prominent in all of the lignins (Figure 4A). Compared to other lignins, CL was weak in A_β_ (C_β_-H_β_ in γ-OH β-O-4, δ_C_/δ_H_ 86.5/4.10), A’_β_ (C_β_-H_β_ in γ-acylated β-O-4, δ_C_/δ_H_ 83.6/4.32), and C_γ_ (C_γ_-H_γ_ in β-β, δ_C_/δ_H_ 71.7/3.81 and 4.17), and there was no adsorption in C_β_ (C_β_-H_β_ in β-β, δ_C_/δ_H_ 53.7/3.12) and B_β_ (C_α_-H_α_ in β-5, δ_C_/δ_H_ 87.7/5.45). PL had no signal for D_α_ (C_α_-H_α_ in β-1, δ_C_/δ_H_ 82.1/5.12) and D_α’_ (C_α’_-H_α’_ in β-1, δ_C_/δ_H_ 85.4/4.80). In the aromatic regions of lignins, cross-signals from H, S, and G lignin units were observed (Figure 4B). The S-lignin units show a prominent signal for the C_2,6_-H_2,6_ correlation at δ_C_/δ_H_ 103.8/6.68, whereas the G units show different correlations for C_2_-H_2_, C_5_-H_5_, and C_6_-H_6_ at δ_C_/δ_H_ 111.5/6.99, 115.2/6.71 and 6.94, and 119.5/6.83, respectively. The C_2,6_-H_2,6_ aromatic correlations from H units (δ_C_/δ_H_ 128.2/7.17) were significantly observed in CL, had weak signals in KL, and had trace content in AL and PL, in accordance with the FTIR analysis. The A”_α’_ (C_α’_-H_α’_ in *p*-coumaroylated substructures) and A”_β’_ (C_β’_-H_β’_ in *p*-coumaroylated substructures) are clearly observed only in CL, in accordance with the FTIR and ^13^C-NMR analysis, also further supporting the GSH-type of CL. No adsorption was observed at any signal for S units and main adsorption at any signal for G units, which also further supported the G-type for PL, also in accordance with the FTIR analysis.

**Table 4:** **Assignment of **^**13**^**C-**^**1**^**H cross-signals in the HSQC spectra of lignin**

**Labels**	δ_**C**_**/**δ_**H **_**(ppm)**	**Assignments**
C_β_	53.7/3.12	C_β_-H_β_ in β-β (resinol) substructures (C)
D_β_	56.1/3.09	C_β_-H_β_ in β-1 (spirodienone) substructures (D)
-OCH_3_	57.3/3.77	C-H in methoxyls
Aγ	60.0/3.38 to 3.71	Cγ-Hγ in β-O-4 substructures (A)
A’γ	63.8/3.83 to 4.30	Cγ-Hγ in γ-acylated β-O-4′ substructures (A’ and A”)
Cγ	71.7/3.81 and 4.17	Cγ-Hγ in β-β (resinol) substructures (C)
Aα/A’α	72.3/4.86	Cα-Hα in β-O-4 substructures (A, A’, and A”)
Dα	82.1/5.12	Cα-Hα in β-1 (spirodienone) substructures (D)
A’_β_	83.6/4.32	C_β_-H_β_ in γ-acylated β-O-4 substructures (A’ and A”)
Cα	85.4/4.64	Cα-Hα in β-β (resinol) substructures (C)
Dα_’_	85.4/4.80	Cα_’_-Hα_’_ in β-1 (spirodienone) substructures (D)
A_β_	86.5/4.10	C_β_-H_β_ in γ-OH β-O-4 substructures (A)
Bα	87.7/5.45	Cα-Hα in β-5 (phenylcoumaran) substructures (B)
S_2,6_	103.8/6.68	C_2_-H_2_ and C_6_-H_6_ in etherified syringyl units
S_2,6_ (Cα = O)	106.7/7.36 and 7.21	C_2_-H_2_ and C_6_-H_6_ in oxidized (Cα = O) syringyl units
G_2_	111.5/6.99	C_2_-H_2_ in guaiacyl units
D_2’_	111.6/6.23	C_2’_- H_2’_ in β-1 (spirodienone) substructures (D)
A”_β’_	114.3/6.24	C_β’_-H_β’_ in *p*-coumaroylated substructures (A”)
G_5_	115.2/6.71 and 6.94	C_5_-H_5_ in guaiacyl units
A”_3’_,_5’_	116.2/6.77	C_3’_- H_3’_ and C_5’_- H_5’_ in *p*-coumaroylated substructures (A”)
D_6’_	118.3/6.19	C_6’_- H_6’_ in β-1 (spirodienone) substructures (D)
G_6_	119.5/6.83	C_6_-H_6_ in guaiacyl units
H_2,6_	128.2/7.17	C_2_-H_2_ and C_6_-H_6_ in *p*-hydroxyphenyl units
A”_2’_,_6’_	130.5/7.4	C_2’_- H_2’_ and C_6’_- H_6’_ in *p*-coumaroylated substructures (A”)
A”α_’_	145.1/7.39	Cα_’_-Hα_’_ in *p*-coumaroylated substructures (A”)

#### *Lignin composition*

Based on the number of carbons per aromatic ring of the C-2 of G units, C-2/C-6 of S units, and C-4 of H units in ^13^C-NMR [16], the S/G ratios were established to be 2.89, 0.87, 1.67, and 0 for KL, CL, AL, and PL, respectively. S/G/H ratio was 0.62:0.71:1 for CL, in accordance with the FTIR and NMR analysis that shows that H lignin was abundant. Even though kenaf is a herbaceous plant, the analysis shows that KL tended to have the hardwood lignin (GS-type), which was demonstrated by the high S/G ratio, the absence of signal for *p*-coumaric acid in ^13^C-NMR and 2D-HSQC NMR, and the low content of H units in FTIR and NMR analyses. Previous analysis from FTIR showed that the low S/G ratio was the main factor of the significant difference in the adsorption of enzyme onto ACL and ASL, and G lignin had higher adsorption capacity on protein than S units. Moreover, according to the results of adsorption and the analysis of S/G ratio in lignins, S/G ratio had a good negative correlation between S/G ratio and adsorbed protein content (*R*^2^ = 0.94), which means that low S/G ratio could be confirmed as one factor of the high adsorption capacity for lignin. Figure 4 shows that the amount of H units decreased in the order CL > KL > PL > AL. The almost equivalent amount of H units in PL and AL, and the greater adsorption capacity of PL than AL indicate that the concentration of the H unit in lignin did not affect the adsorption capacity. CL had more H units and weaker affinity than PL, and KL had more H units and weaker adsorption capacity than AL and PL, indicating that the increase in the amount of H units did not increase the adsorption capacity. CL had more H units and greater affinity than KL, demonstrating that the increase in the amount of H units did not decrease the affinity. These results show that the amount of H units may not be related to the adsorption capacity.

Even though the influence of altering the S/G ratio of the moieties in lignin on the chemical and physical properties of the cell wall and glucose yield is being actively studied [4,5,17], there are no studies about the effect of the amount of the H unit on the enzymatic digestibility of the biomass and the three lignin monomers on adsorption capacity until now. In the enzymatic hydrolysis, *Arabidopsis* mutant 063792 not only presented higher cellulose conversion compared to *Arabidopsis* wild-type Col-0, whether with high or low enzyme dosage (data not shown), but also increased cellulose conversion much higher with low enzyme dosage (48%) than with high enzyme dosage (14%), although there is almost same content of total lignin in the two *Arabidopsis* lines. This result further demonstrates that the low S/G ratio produced high glucose yield for untreated samples [5], and S units rendered more resistance to degradation by cellulase [18], which more significantly appeared with low enzyme dosage and sufficiently counteracted the lower adsorption on cellulase. Papa *et al*. [5] reported that high lignin-S/G ratio produced low glucose yields for untreated samples (*r* = -0.97; *P* <0.03; n = 4), but the alteration of lignin-S/G ratio did not affect the glucose yield after ionic liquid (IL) pretreatment, which is attributed to the high efficacy of IL pretreatment that masked the effect of the altered S/G ratios. The S/G ratio has also been reported to affect enzymatic hydrolysis because it affects lignin cross-linking, and thus, the three-dimensional structure of the plant cell wall and enzyme accessibility [17]. The chemical composition and the structural elements of lignin are regarded as barriers to enzymatic hydrolysis [19]. The downregulation of S units also leads to the incorporation of novel units into the lignin that arises from the intermediates in the lignin synthesis pathway [20], such as 5-hydroxyconiferyl alcohol. The inclusion of these novel intermediates may ‘loosen’ the overall lignin structure and are responsible for improving its digestibility. Pan [9] also stated that the concentration of lignin methoxyl groups is highly negatively correlated with the degradation of the cell wall, and believed that the high likelihood of quinone methide formation is the reason for the greater inhibitory effect of S units than G units.

#### *Molecular weight determination*

The weight average molecular weights (Mw), number average molecular weights (Mn), and PDI (Mw/Mn) of the lignins were determined using GPC analysis (Table 5). The average molecular weight of the lignins from the different types of lignocellulosics and PDI were difficult to relate to protein binding, which was also reported in other studies [6]. Nevertheless, GPC showed that PL and CL had more uniform lignin fragment size than AL and KL, which is favorable for the interaction with proteins by higher plasticity [1]. The uniformity in lignin fragment size decreased in the order PL, CL > AL > KL, which is in accordance with the order of adsorption capacity.

**Table 5:** Molecular weights and polydispersity index of lignins

**Lignin sample**	**Mn (Da)**	**Mw (Da)**	**Polydispersity (Mw/Mn)**
KL	36,719	147,755	4.02
CL	44,530	163,802	3.68
AL	47,389	224,615	4.74
PL	20,039	78,149	3.90

AL, aspen lignin; CL, corn stover lignin; KL, kenaf lignin; Mn, number average molecular weights; Mw, weight average molecular weights; PL, pine lignin.

## Conclusions

Through the comparison of the protein adsorptions onto six lignins and the examination of the properties of the lignins, lignin sources were found to affect enzyme adsorption through their lignin composition and structural features, like functional groups, such as carboxylic acid, aliphatic hydroxyl, and phenolic hydroxyl groups. The adsorption capacity decreased in the order PL > CL > AL > KL. The lower the S/G ratio, the higher affinity was observed. For mono-component enzymes, the adsorption capacity decreased in the order CBH, xylanase > EG > BG. The investigations in this paper not only reveal the differences of lignin properties among diverse biomass, the adsorption capacity of the enzymes to lignins, and its possible mechanism, but also provides the reference for the genetic modification of lignocellulosic biomass and enzymes.

## Methods

### Microorganism and biomass materials

*P. oxalicum* JUA10-T, a mutant from *Pcp oxalicum* JU-A10, was stored in the laboratory. The medium composition for cellulase production was based on that previously described [21].

Kenaf stalk and corn stover were obtained from Xinjiang Uygur Autonomous Region and Shandong Province, China, respectively, and aspen and pine (*Pinus massoniana* Lamb.) were provided by Tianjin University of Science and Technology, Tianjin, China. *Arabidopsis* Col-0 (wild-type) and *Arabidopsis* mutant 063792 (SALK mutant of *fah1*) were generously granted by the Botany Laboratory at Shandong University, Shandong Province, China.

### Preparation of lignins, holocellulose, and PASC

The MWL from kenaf, corn stover, aspen, pine (*P. massoniana* Lamb.), *Arabidopsis* Col-0, and *Arabidopsis* SALK mutant 063792 in this study were isolated from lignocelluloses according to the description in the literature [22]. MWL is a lignin preparation considered to be the most representative of the whole native lignin in the plant [22], and is mainly used to study the structure of lignin [10,16] despite its low yield. However, the isolated MWL is the only lignin fraction that was extracted using aqueous dioxane (96%) solution, and did not represent the structure and characteristics of all the lignin in the plant. The holocellulose from *Arabidopsis* Col-0 and PASC (1%, w/v) were prepared according to the description by Eom *et al*. [23] and Zhang *et al*. [24], respectively.

### Enzyme adsorption

The adsorption of the enzyme preparation to lignins was performed in polypropylene tubes (2 mL) using 3.3% (w/v) lignin and 5.4 mg g^-1^ lignin of protein in 50 mM sodium acetate buffer (pH 4.8) at 45°C for 48 h in a rotary shaker (QB-228, Kylin-Bell Lab Instruments Co., Ltd, Haimen, Jiangsu Province, China). Lignins appeared to be insoluble. Samples were centrifuged (10,000 rpm, 10 min) and the liquid containing the unbound enzymes was collected (adsorption supernatant) for further analysis. Controls lacking lignin or enzyme were used as reference.

### Protein analysis and enzyme assays

Protein concentrations were determined using the method described by Bradford [25]. The activities of CBH, EG, BG, and xylanase were assayed using 1% *p*NPC, CMC-Na, salicin, and xylose (Sigma-Aldrich, St Louis, MO, USA) as the substrate at pH 4.8 (50 mM sodium acetate buffer) at 50°C for 30 min according to the procedures of Gao *et al*. [26] and Guo *et al*. [27]. One unit (U) of enzyme activity was defined as the amount of enzyme that liberated 1 μmol of reducing sugar per minute under the assay conditions.

### Enzymatic hydrolysis

The hydrolysis tests for Avicel (Sigma-Aldrich), holocellulose, and PASC were conducted using the enzyme and each lignin (3.3%, 5.4 mg protein g^-1^ lignin) at 45°C for 48 h in a rotary shaker (QB-228). The substrate for loading was double lignin. In the supernatant, glucose and xylose were determined using HPLC (Shimadzu, Kyoto, Japan) with a refractive index detector (Shimadzu) on an Aminex HPX-87P column (Bio-Rad, Hercules, CA, USA) at a flow rate of 0.5 mL/min at 78°C, with water as the eluent. Samples lacking lignin or enzyme served as control.

### Lignin characterization

The functional groups of the isolated lignins were analyzed using FTIR (Nexus, Thermo Nicolet, Thermo Fisher Scientific, Waltham, MA, USA), with KBr pellets, over the range of 400 to 4,000 cm^-1^, and NMR spectra with an Avance 400 MHz spectrometer (Bruker, Billerica, MA, USA) at 25°C in DMSO-d6. For ^1^H-NMR, 10 mg of lignin was dissolved in 0.5 mL DMSO-d_6_, and 100 mg of lignin for ^13^C-NMR and 2D-HSQC spectra. The sequence was conducted according to literature [10].

The molecular weights of the lignins were determined using GPC with dimethylformamide (DMF) [6].

## Abbreviations

ACL: *Arabidopsis* Col-0 lignin; AL: Aspen lignin; ASL: *Arabidopsis* SALK mutant 063792 lignin; BG: β-glucosidase; CA: *p*-coumaric acid; CBH: Cellobiohydrolase; CBM: Carbohydrate-binding module; CL: Corn stover lignin; DMF: Dimethylformamide; DMSO: Dimethyl sulfoxide; DMSO-d6: Deuterated dimethyl sulfoxide; EG: Endoglucanase; F5H: Ferulate-5-hydroxylase; FA: Ferulic acid; FTIR: Fourier transform infrared spectroscopy; G: Guaiacyl; GPC: Gel permeation chromatography; H: Hydroxyphenyl; HPLC: High performance liquid chromatography; HSQC: Heteronuclear single quantum coherence; IL: Ionic liquid; KL: Kenaf lignin; Mn: Number average molecular weights; Mw: Weight average molecular weights; MWL: Milled wood lignins; NMR: Nuclear magnetic resonance; PASC: Phosphoric acid-swollen cellulose; PDI: Polydispersity index; PL: Pine lignin; S: Syringyl.

## Competing interests

The authors declare that they have no competing interests.

## Authors’ contributions

FFG performed the experiments, data analysis, and drafted the manuscript. WJS, WS, and FFW carried out the experiments, data analysis, and contributed to the manuscript draft. XZL, JZ, and YBQ designed the project, critically analyzed the data, and revised the manuscript. All authors read and approved the final manuscript.
